# 

**Published:** 1972-01

**Authors:** 


					Bristol Medico-Chirurgical Journal Vol. 87 (i) No. 321
x,
Contents
Glaucoma
C. A. Brown
The Prevention of Bleeding During Long-Term Oral
Anticoagulant Therapy
R. D. Eastham
Obituary
Medical Library   10
Society Reports   13
F. Bailey & Son Ltd., Dursley, Glos.

				

